# Developing Typologies of User Engagement With the BRANCH Alcohol-Harm Reduction Smartphone App: Qualitative Study

**DOI:** 10.2196/11692

**Published:** 2018-12-13

**Authors:** Joanna Milward, Paolo Deluca, Colin Drummond, Andreas Kimergård

**Affiliations:** 1 Addictions Department King's College London London United Kingdom

**Keywords:** young adults, binge drinking, drinking, smartphone, mobile phone, mHealth

## Abstract

**Background:**

Understanding how users engage with electronic screening and brief intervention (eSBI) is a critical research objective to improve effectiveness of app-based interventions to reduce harmful alcohol consumption. Although quantitative measures of engagement provide a strong indicator of how the user engages with an app at the group level, they do not elucidate finer-grained details of how apps function from an individual, experiential perspective and why, or how, users engage with an intervention in a particular manner.

**Objective:**

The aim of this study was to (1) understand why and how participants engaged with the BRANCH app, (2) explore facilitators and barriers to engagement with app features, (3) explore how the BRANCH app impacted drinking behavior, (4) use these data to identify typologies of users of the BRANCH app in terms of engagement behaviors, and (5) identify future eSBI app design implications.

**Methods:**

In total, 20 one-to-one semistructured telephone interviews were conducted with participants recruited from a randomized controlled trial, which evaluated the effectiveness of engagement-promoting strategies in the BRANCH app targeting harmful drinking in young adults (aged 18-30 years). The topic guide explored users’ current engagement levels with existing health promotion apps, their views toward the effectiveness of such apps, and what they liked and disliked about BRANCH, specifically focusing on how they engaged with the app. Framework analysis was used to develop typologies of user engagement.

**Results:**

The study identified 3 typologies of engagers. *Trackers* were defined by their motivations to use health-tracking apps to monitor and understand quantified self-data. They did not have intentions necessarily to cut down and predominantly used only the drinking diary. *Cut-downers* were motivated to use the app because they wanted to reduce their alcohol consumption Unlike *Trackers*, they did not use a range of different health apps daily, but saw the BRANCH app as an opportunity to test out a different method of trying to cut down their alcohol use. This typology used more features than Trackers, such as the goal setting function. *Noncommitters* were characterized as a group of users who were initially enthusiastic about using the app; however, this enthusiasm quickly waned and they gained no benefit from it.

**Conclusions:**

This was the first study to identify typologies of user engagement with eSBI apps. Although in need of replication, it provides a first step in understanding independent categories of eSBI users, who may benefit from apps tailored to a user’s typology or motivation. It also provides new evidence to suggest that apps may be used more effectively as a tool to raise awareness of drinking, instead of reducing alcohol use, and be a step in the care pathway, identifying at-risk individuals and signposting them to more intensive treatment.

**Trial Registration:**

International Standard Randomised Controlled Trial Number ISRCTN70980706; http://www.isrctn.com /ISRCTN70980706 (Archived by WebCite at http://www.webcitation.org/73vfDXYEZ)

## Introduction

### Background

User engagement rates with app-based electronic and screening interventions (eSBI) are consistently reported as lower than expected in app-based eSBI trials [1,2]. A recent study of the Drinkaware app demonstrated that less than 50% of those who downloaded the app used it for more than a week, and only 14% were using it after a month [1]. Engagement with eSBI is critical because studies demonstrate an association between the level of a user’s engagement and the effectiveness of the Web-based behavior change intervention [1,3-5]. Understanding how users engage with eSBI is a key research objective to improve effectiveness of app-based interventions to reduce harmful alcohol consumption [6-8].

User engagement with eSBI apps is a complex and multifaceted behavior, which requires nuanced exploration of the subtleties of how individuals interact with the electronic health (eHealth) intervention [9-12]. Engagement has been conceptualized in different ways, both in terms of how the user interacts with the technology as well as how the user experiences it [9]. The interaction may be simple such as visiting a particular feature of the eHealth intervention or completing a more complex task such as filling out a diary. Engagement can include how long or how often the participant uses the eHealth intervention [13,14].

Engagement has also been defined qualitatively. From this perspective, it is characterized as a *state* of involvement with the technology, such as becoming fully absorbed in the process. Outside of the alcohol field, research has examined *typologies* or *trajectories* of user engagement that enable distinctions to be made between different types of user behavior, which has the potential to target more tailored interventions to users [15]. Yardley et al [12] make a useful distinction of conceptualizing engagement at the micro and macro level. The micro level reflects the moment-to-moment interactions that occur as a user engages with features of the technology, whereas the macro-level engagement refers to how the user engages with the overall behavior change goal.

Although quantitative measures of log-ins or page visits measured in previous eSBI app trials provide a strong indicator of how the user engaged with the app at a group level, they neither elucidate finer-grained details of aspects of why users engaged with the app in a particular manner nor provide information on features that worked well, and those that did not, from an individual experiential perspective [9-12].

A qualitative exploration allows for examination of engagement, which puts the characteristics of the individual user experiences as the primary focus, and helps to contextualize the findings of the quantitative usage patterns to understand why users engaged as they did. [11,12]. It is argued that measures of engagement that rely purely on usage data do not provide a comprehensive measure of the subtleties of engagement behavior [11,12,15]. For example, quantitative measures of feature usage may identify those that were most frequently used, but not why they were used more than others, or what impact they had. Therefore, qualitative investigations of engagement are required to fill this gap in the existing knowledge of engagement.

There are various models of user engagement with digital interventions. O’Brien and Toms’ [16] well-established conceptual model of user engagement (CMUE) is distinguishably qualitative than other models as they conceptualize engagement as a *quality of user experiences* characterized by a series of attributes such as esthetics and novelty. They summarize these attributes comprising *threads of experience* through the different stages of engagement. The *threads of experience* comprise sensual, emotional, and spatiotemporal categories, which are particularly useful in conceptualizing engagement from a qualitative perspective. Sensual experience refers to esthetic or novel elements, which promote attention and interest to the program, and emotional experience refers to the positive or negative affect elicited from the program. Finally, spatiotemporal experiences refer to the perceptions and awareness of time and physical surroundings the user feels when using the program, for example, experiencing a state of *flow* or the fast passing of time.

Short et al [17] propose the first evidence-informed model, specifically dedicated to the issue of engagement with eHealth interventions. The model considers the relationships between the individual (user characteristics), environment (social and physical characteristics), and intervention-level factors (content and features) that contribute to a user’s engagement with an eHealth intervention. At the core of the model is the concept of tailoring interventions, with the aim of producing an intervention that is relevant, novel, appealing, and motivating to the user. According to the Elaboration Likelihood Model, people are more motivated to process information elaborately, leading to long-lasting effects, if the message is personally relevant or *tailored* to them [17]. The model brings together theory from O’Brien and Toms’ [16] CMUE as well as Oinas-Kukkonen and Harijuanas’ persuasive systems design (PSD) [17] (see Ryan et al [10] for a comprehensive scoping review of engagement theory).

Surprisingly, examination of engagement within the eSBI field from a qualitative research perspective is somewhat limited. Quantitative measures of engagement, such as log-ins or time spent on the Web, are more typical across the literature, and qualitative measures have been largely ignored. Studies have examined *usability* with a qualitative research design in the alcohol field [6,18]; however, *usability* typically refers to a single factor that influences engagement at the interaction level with the program and does not encompass the full breadth of elements that comprise engagement [9,10,12,19].

A few studies from the wider health care literature have directly explored engagement qualitatively, for example, in the smoking cessation and weight loss fields [15,20]. The most comprehensive study is by Smith et al [15] who examined trajectories of engagement and disengagement for a *story-based* smoking cessation app with an interview-based method. The app presented quitters’ stories and allowed users to read and post content. They proposed a new *conceptual trajectories’* model of different types of engagement, ranging from productive to counterproductive engagement as well as productive and nonproductive disengagement. Productive engagement is the desired state of use where users are highly invested in the smoking cessation tool and fully identify with the program components. Counterproductive engagement and nonproductive disengagement referred to experiences that had an effect opposite to that which was intended such as antipathy toward the program and even increases in smoking level. Productive disengagement, on the other hand, occurred when users positively engaged with the intervention and then went on to make a quit attempt. The trajectories model has implications in terms of how disengagement is considered and the level of control a user should be given over how they use the technology. Of particular note is that disengagement is not necessarily always an undesired behavior, and users should be free to disengage with the app if they feel it has had a positive impact. The model has, however, only been examined with a smoking cessation population, and although potentially applicable across other substances such as alcohol, the model has not been applied across drug and alcohol substances.

As is evident, few studies have qualitatively explored engagement with health apps, and none has comprehensively examined engagement with a qualitative research design with eSBI apps. In addition to the many quantitative studies exploring engagement using simple server and usage data, rich and detailed explorations of engagement from a qualitative perspective, such as the trajectories study by Smith et al [15] described above, are required to meaningfully understand the complexities and subtleties that engagement encompasses and elucidate insights and contextual factors that are beyond the abilities of a purely quantitative design.

Developing qualitative *typologies* of user engagement enhances existing quantitative typologies developed in the broader health behavior change field and contributes to the literature aiming to tailor apps to specific user types, which is a well-established engagement-promoting technique.

### Objectives

The aim was to explore participants’ experiences of engaging with the BRANCH app over a 28-day period to (1) understand why and how participants engaged with the BRANCH app, (2) understand facilitators and barriers to engagement with the app features and intervention goals, (3) explore how the BRANCH app may have impacted drinking behavior, (4) identify from these data different typologies of users in terms of engagement behaviors, and (5) identify future eSBI app design implications.

## Methods

### Study Context

This qualitative study was part of a larger research project, which developed an eSBI smartphone app (called BRANCH), aimed at promoting engagement, targeting harmful drinking in young adults, and evaluating it with a mixed-methods approach including both an randomized controlled trial (RCT) and qualitative interviews with trial participants. The qualitative study is the focus of this paper. For context, the RCT evaluated the effectiveness of a comprehensive version of the BRANCH app, including novel, innovative engagement-promoting strategies (EPSs, intervention arm) to increase app engagement as measured by user log-ins compared with a basic version (control arm), including minimal EPSs, which included only established screening and brief intervention techniques [21]. The RCT (Milward et al, in preparation) also measured change in harmful drinking between arms. See the following publications for further detail on the BRANCH app and a description of the development phases [6-8].

### Design

One-on-one semistructured telephone interviews were conducted to explore individual user experiences with the app in the context of their daily lives. Individual interviews provided the opportunity for an in-depth understanding of the individual perspective around which the research objectives were situated [22]. In addition, exploration of how participants used the app to monitor their own drinking was more appropriately conducted in a one-on-one interview design compared with a focus group setting to minimize the potential for participants feeling uncomfortable about discussing sensitive topics about harmful drinking.

### Sample

A purposive sample of 20 participants who took part in the BRANCH app RCT was recruited, with approximately equal numbers of high and low engagers from either the comprehensive or basic app group. On the basis of the analysis methods outlined in the protocol of the RCT (ISRCTN registration number 70980706), high engagers were classified as having logged in more than once and low engager as having logged in once. Sampling for interviews occurred across the first 3 months of recruitment in the RCT (January 2017 to March 2017).

In total, 20 participants were recruited to achieve a level of representational generalization [22] to uncover the breadth and nature of the views and experiences of the participants, which reflected those of the wider population from the RCT. In qualitative research, generalization cannot be achieved statistically but instead in terms of reaching saturation in the data [22]. Moreover, 20 participants were also selected as this would include 10 high engagers and 10 low engagers across both trial arms.

### Recruitment

Participants were considered eligible 28 days after randomization into the trial so as to not bias the primary outcome of the RCT, which was collected at day 28 post randomization. Participants’ details from the RCT were extracted from the Web-based trial management system. Participants were categorized by app version (comprehensive vs basic) and whether they were high or low engagers. Eligible participants were contacted by email in blocks of 20, stratified by month of RCT recruitment and engagement type. Blocks were selected by date, starting with participants randomized on the first day of the month. Interviews were allocated on a first-come-first-serve basis. If no interviews were completed after the first block, consecutive blocks of 20 participants were emailed until the quota for each month and engagement level were achieved. Of the 211 RCT participants who were emailed, 30 replied requesting for an interview.

### Description of the BRANCH App

The BRANCH app was developed in 3 stages: a systematic review of EPSs for Web-based substance use interventions [7]; scoping focus groups to determine user preferences for content, features, and style [8]; and usability testing on the prototype BRANCH app with a sample of the target population [6]. From the systematic review, 3 EPSs were identified that may promote engagement, tailoring, reminders, and delivery strategies. From the scoping focus groups, 2 main themes were identified. The *meaningfulness* theme reflected how young adults thought apps needed to be tailored to the interests and values of their age group, particularly emphasizing on content and feedback about broader health and well-being factors such as exercise, diet, and image. The *community* theme suggested that young adults wanted to be able to engage with other app users both in groups of friends and with Web-based users for motivation and support. From the usability testing, an easy-to-use interface with minimum required user-input was a critical usability issue for young adults. Clear, consistent, and visually appealing design was integral to the level of usability. The option for social connectivity was important, as were the high levels of personalization. Poor functionality was considered a major usability barrier.

The core alcohol harm reduction components of BRANCH were based on the Feedback, Responsibility, Advice, Menu of options, Empathy, Self-efficacy (FRAMES) model of alcohol brief interventions [21], which has been previously adapted for eSBI [23]. The FRAMES model is based on the principles of motivational interviewing, an established and evidence-based method to reduce alcohol-harm [24]. Core functions, which constituted the basic (control) version of BRANCH, included a drinking diary for recording of alcohol consumption and a goal setting function where users could set weekly goals based on alcohol units as well as setting a drink-free day. Users could monitor their drinking over time and receive feedback on it both descriptively and graphically. Information on drinking risks and benefits of cutting down was available to users.

The *comprehensive* (intervention) version of BRANCH included a number of targeted EPSs. Several theoretical models were used, including O’Brien and Toms’ CMUE as described above and Oinas-Kukkonen and Harijuanas’ PSD [17]. The comprehensive version included a Twitter or Facebook style newsfeed enabling interaction between app users, as well as providing tailored notifications, motivational messaging (including positive reinforcement and praise), and in-app reminders based on goals. The research team could also upload relevant material such as links to news articles, YouTube videos, and photographs. BRANCH was tailored; when signing up for the app, users selected their motivations for cutting down drinking. Personalized feedback and tailored information were delivered to users based on their selection of motivators. Additional goals were included such as reducing sugar, calories, and money spent on alcohol. Participants were encouraged to set goals based on their selected motivator. Extended infographic-style information targeted to motivations for cutting down were included. Users were allocated to a team based on these motivators. A *teams* page with a separate newsfeed channel was available for each motivator. Users could compare their progress against other users in their team as well as between teams and were awarded points for engaging with the app in line with gamification principles. Content was provided in a multimedia format, with a single exposure of content (all at once as opposed to staged).

BRANCH was a Web-based app, which meant it was hosted on the Web and on a server instead of being on a user’s phone. For users, this meant that the app was not accessed and downloaded from the app store but was logged into the Web at each point of use.

### Ethical Approval

Ethical approval was obtained from the University Research Ethics Committee (reference number RESCMR-16 or 17-2896).

### Data Collection

Eligible participants were invited to participate in a semistructured interview lasting between 30 and 60 min and conducted by telephone. They were provided with an information sheet via email. On completion, participants were reimbursed with a £20 voucher for their time.

Consent was audio-recorded at point of interview. Semistructured interviews explored users’ current engagement levels with health promotion apps, their views toward the effectiveness of such apps, and what they liked and disliked about the BRANCH app, specifically focusing on how they engaged with the app. The questions were open ended, encouraging participants to share as much information as possible about their experiences. The topic guide was broken into stages of engagement, drawn from O’Brien and Toms’ CMUE [16], asking participants to describe their experience at each stage of engagement, for example, their motivations for first using the app, first use, and ongoing and disengagement experiences. The topic guide was tailored to arm allocation and user engagement level (Multimedia Appendix 1).

### Reflexivity

The first author conducted all the interviews and had significant experience of moderating both 1:1 and focus group interviews. She had no previous relationship with any of the participants before the interview other than email contact to set up the interview. The only information participants were given about the interviewer was that they were a member of the app research team. In terms of reflexivity, the first author developed the app herself; therefore, special attention was paid to reduce social desirability bias in the interviews, whereby a positive response may have been elicited from the participants to be supportive of the author’s work. To overcome this bias, participants were not told that it was the interviewer who developed the app and were encouraged to provide both positive and negative feedback.

### Data Analysis

The interviews were recorded, and data were transcribed using a professional transcription service. Transcripts were checked for accuracy against the recordings, and names as well as other personally identifiable data were changed. The data were coded by the first author using QSR International’s NVivo version 11.4.1 software. A Framework approach was used to analyze the data [22]. Framework analysis was developed to support the aims of applied research, such as mapping out the range and nature of a phenomenon, finding linkages and patterns within data, and creating typologies of behaviors or attitudes. Its objectives are to meet specific needs with actionable outcomes such as evaluating an intervention. Its key features are that it is grounded deeply in the accounts of the participant and that it is systematic, comprehensive, and allows for cross-case and between-case analysis. A framework matrix was created comprising descriptive themes and subthemes for each individual participant (or case). Specifically, the framework analysis comprised 5 key steps. First, the researcher read through the raw transcripts to gain familiarity with the data and create an initial map of themes derived from the research questions and identified themes from the data. Second, the researcher used this initial thematic framework to code (or index) the raw data using NVivo and indexed the raw data extracts into their relevant thematic category. The thematic framework was then reviewed and refined to see if all important themes and subthemes were covered and were practically distinguishable from one another. Next, the data were summarized into a framework matrix to reduce the data for later thematic abstraction. This was completed in the data processing tool, Microsoft Excel. Each theme was given its own matrix, with each subtheme allocated a column and each participant (or case) a row. For each theme, the researcher summarized the raw data coded at each of the subthemes, keeping as close to the data as possible using key terms, expressions, or phrases from the participants.

The final stage was the abstraction and interpretation of the summarized data. For each theme and subtheme for each case, the researcher read all the summaries and noted key elements, perceptions, and views in an additional column inserted into an Excel spreadsheet. From these underlying dimensions, higher-order categories were developed and abstracted from the data. This process moved the data from the descriptive to the more conceptual level. From these abstracted data, linkages were developed: linkages refer to connections or patterns between different sets of phenomena in the data or can also refer to whether there are links between sets of phenomena such as beliefs, experiences, or behaviors and different subgroups in the study—for example, linking together experiences of app usage and disengagement behaviors or understanding the link between app use and impact on drinking. For clarity, thematic frameworks around app engagement were conceptualized according to Short et al’s [19] model of user engagement where engagement was categorized according to individual-level, environmental-level, or app-level engagement factors.

To explore distinct types of engagers, complex typologies were developed involving the interconnection between dimensions such as particular beliefs, experiences, or behaviors (or *positions*) in the data. Multiple-linkage typologies were developed [22], which refer to unique clusters or combinations of *positions* that create distinct typologies. Although the same *position*, such as a specific view, behavior, or belief, can occur across more than 1 typology, it is the unique combinations of positions that create the distinct typologies. Typologies were first developed at the individual case level across the framework matrices and were then abstracted to the phenomena level (as opposed to case level). To check the robustness of the typologies, the researcher went back to the case level to check for fit against each individual case. Finally, to provide recommendations for future app development, higher order findings across all the aims were summarized into key priorities and *meta* findings.

## Results

### Participant Characteristics

A total of 20 participants completed the interview, 11 from the intervention group and 9 from the control group. There were more females (16/20, 80%) than male participants (Table 1). This reflects the overall sample of the RCT where 70% participants were female. The mean age of the sample was 24 years (SD 3.0). Participants were spread over a wide geographic area of the United Kingdom in a range of professions and education. The majority of participants (12/20, 60%) were students, which again was consistent with the overall sample of the RCT. The mean number of log-ins to the app was 8 (SD 10.3), with a range of 1 to 35.

**Table 1:** Participant demographics, allocation in randomized controlled trial (RCT), and use characteristics.

Description of sample		Whole sample	High engagers	Low engagers
Intervention arm, n (%)		11 (55)	6 (30)	5 (25)
Control arm, n (%)		9 (45)	5 (25)	4 (20)
Age in years, mean (SD)		24.0 (3.0)	25 (3.1)	22 (2.2)
Female, n (%)		16 (80)	8 (40)	8 (40)
**Occupation, n (%)**				
	Student	12 (60)	5 (25)	7 (35)
	Employed	7 (35)	5 (25)	2 (10)
	Unemployed	1 (5)	0 (0)	1 (5)
Log-ins, mean (SD)		8.0 (10.3)	15.0 (10.9)	1.0 (0)

### Typologies of User Engager

A principal aim of the analyses was to identify distinct user typologies, of which 3 were identified: the Tracker, the Cut-downer, and the Noncommitter. These typologies are outlined in detail below.

#### The Tracker: Monitoring and Tracking Alcohol Consumption

This was the most common type of app engager. The defining feature of the participants in this group was that their motivations for using the app were not primarily to cut down but mostly to monitor and keep track of their alcohol use. Some were also interested in monitoring their spending and finding out whether or not they were at risk of the harmful effects of drinking. Participants in this group were very conscious about monitoring not only their alcohol usage but also their health and lifestyle in general. Trackers used multiple health apps to track a range of lifestyle factors such as calories, exercise, finances, menstrual cycle, and sleeping patterns. For Trackers, using BRANCH was just an extension of their current health app usage, which fitted easily into their daily habits of entering data into a variety of health apps:

I find it quite satisfying to have it logged down accurately. But I enjoy things like this, like I’ve always kept a diary since I was young, I’ve always used like apps to track my menstrual cycle and always kept up to date with them, and use them really accurately...

Female, 24, high engager

Trackers described themselves as organized individuals who wanted the structure that monitoring can provide to their day-to-day lives. Trackers had a strong positive emotional response to keeping track of their alcohol use, which made them feel in control and empowered.

##### How Trackers Engaged With BRANCH Features

Trackers consisted of both high and low engagers. The high engagers would use the app consistently and meticulously; whereas some used it daily or weekly, others stepped in and out of using it. Low-engaging Trackers typically just put in a few drinks but still identified their motivations to be to track their health as opposed to cutting down. High-engaging Trackers were strongly motivated to use the app but were focused particularly on entering drinks into the drinking diary. Trackers would typically just log in and log out to enter drinks into the drinking diary, ignoring the other features. Trackers had little to say about the other features and were mostly not interested in setting a goal as reduction was not their primary motivation:

I guess what I wanted the app for, for my own exploration of my drinking habits and sort of seeing how much I’m drinking, the drinking diary made the most sense to focus on...I don’t really know what the other stuff does on there because I haven’t, like the goals that I did look at, I haven’t set any goals, I just looked at them...

Female, 20 years, high engager

They reported the information on alcohol presented in the app to be unmeaningful and not relevant to them. This is except for the Information section on units, which allowed them to enter drinks more accurately. In terms of the feedback, Trackers appreciated being able to get feedback on their spending on drinking but did not praise the drinking risk feedback as it was not regularly updated and did not change when they entered their drinking data. Trackers did not use the social features of BRANCH viewing tracking as a solitary activity, not one necessarily shared with others. None of the Trackers used the Team section.

##### Impact on Drinking for Trackers

The majority of Trackers did not have intentions to cut down their alcohol use. Some discussed how they thought they might drink too much, particularly in a social context, and viewed the app as an opportunity to find out whether they were drinking at a harmful level. In terms of impact on drinking, they mostly described being made more aware of their drinking patterns and habits, particularly about drinking more mindfully. Some learned that they drank more alcohol than they thought they did. Having different options to visualize the data in different graphs was considered a helpful way to understand drinking habits. A few did describe cutting down, being more motivated to turn down a drink when offered when out with friends, and making more sensible choices when choosing a drink since using the app. However, overall, the app’s impact on drinking was described as increased awareness and not significant change in drinking level:

I wouldn’t say it has had a massive impact. I would say it has made me a lot more aware of how much I drink when I drink. I don’t drink as frequently as other people but I probably drink more than some people do when I do drink. I think that has made me a lot more aware of that, the amount that I drink at one time and so I have been making a conscious effort to kind of restrict that.

Male, 21 years, high engager

##### Facilitators to Use for Trackers

At the app level, engagement facilitators centered around the provision of different features to monitor drinking, costs and calories, and different ways of viewing these data. Accuracy of data was key to Trackers, as was ease of use to enter drinks into the drinking diary such as autofill cues to enter data. For Trackers, any function that made entering drinks faster and more functional was a facilitator to engagement with BRANCH. At the individual level, the provision of monitoring features for this group led to strong positive emotional responses to the app, which fostered motivation to use it. For example, it triggered feelings of control, security, health, empowerment, and autonomy.

##### Barriers to Use for Trackers

At the app level, usability issues such as Web-based app access problems and nonfamiliarity with Web-based apps in general were frequently highlighted and were the main reasons low-engaging Trackers cited for low engagement. Registration problems and length of time taken to enter data were also barriers to engagement for Trackers. However, Trackers were also likely to try to overcome any registration or usability issues when they occurred because of higher levels of motivation to use the app. Although not familiar with Web-based apps, they would work out on how to either pin the icon to their home screen or save the Web page link. A lack of short message service (SMS) text messaging or push notification reminders meant that Trackers sometimes forgot to enter data. Trackers were wary of the privacy issues about the social component: cutting down drinking was considered somewhat a nonacceptable social activity, unlike, for example, fitness trends, or weight loss, which often share data via apps. At the environmental level, barriers to engagement included life constraints such as tiredness, time taken to add in data, being busy at work, or being away on a holiday.

#### The Cut-Downer: Intention to Reduce Alcohol Consumption

The Cut-downers’ primary reason for using the BRANCH app was to reduce their alcohol intake. Participants from this group were worried about the health risks associated with alcohol use and would typically believe that they already drank too much:

Interviewer: And did you want to cut down your drinking at all, was that in your mind?

Interviewee: Yes, definitely, because I know when I was like 17, 18, 19, 20, 21, I used to binge drink, like a lot, so it’d be like nearly every weekend, if not like twice a week, and like as I’ve got older, I’ve cut back on it but this has helped me see, like how much I have cut back and where it is I still need to carry on trying to cut back.

Female, 25 years, high engager

Alternatively, they might have wanted to cut down for other reasons such as those of a financial nature. A few of them had previous quit attempts. Unlike Trackers, Cut-downers were not interested in monitoring all aspects of their health but were predominantly focused on alcohol use. Cut-downers did not use many different health apps but may have tried 1 or 2 in the past, with no regular use.

##### How Cut-Downers Engaged With BRANCH Features

Similar to Trackers, Cut-downers were also very focused on the monitoring features where they could record their alcohol intake and set goals for themselves (see matrix, Multimedia Appendix 2, for a comparison of typology characteristics):

What I mainly used from the app was the drinking diary and the goals. So, the goals of, say, if I was having five or six pints a week, maybe cutting that down to four, something like that...I think the drinking diary helped as well. I filled that in as I was going along.

Female, 26 years, high engager

Cut-downers were all high engagers and typically used the app consistently either daily or at least two to three times per week. Cut-downers were also concerned about the importance of being able to accurately input drinks in the app, particularly about having enough options for brands of drinks and how to record drinks when not pouring or buying one’s own. Cut-downers also had a broader interest in other features of BRANCH, which helped them achieve their goal of cutting down their drinking. Cut-downers were more likely to set a goal than other typologies, with the majority setting at least one goal or a drink-free day. One Cut-downer set a goal for himself without the support of the app, as he did not feel he needed the app to help him with it, suggesting that using the app might encourage behavior change beyond the support offered in the app’s features.

Cut-downers also engaged with the feedback section; they found the feedback on drinking and risks not only eye opening and often shocking but also frustrating as this did not change week to week. One Cut-downer commented on the Newsfeed, but no other participants used the social features stating that it was either not relevant to their goals or, similar to the Trackers, that cutting down on alcohol use is a solitary activity. Concerns were shared over privacy issues of the social component. No Cut-downers used the Team section.

##### Impact on Drinking for Cut-Downers

One participant reported having stopped drinking for a week, and another reported having cut back during the time they were using the app. Cut-downers did not express regret when they did not achieve their goals; instead, similar to the Trackers, they reported an increase in awareness of the amount of alcohol they were consuming as opposed to reduced consumption. This was expressed as a positive outcome and an achievement in itself. Cut-downers also reported being surprised by how much they were actually drinking, about how many units there are in alcohol, and the associated risks:

I think its helped in the fact that I now know what I’m drinking and how much I’m drinking. However, it’s not really helped me cut down as such, because I’m surrounded by it all the time. I’m still trying to cut down.

Female, 22 years, low engager

##### Facilitators to Use for Cut-Downers

Ease of use was cited as a facilitator to using the app. Some Cut-downers favored the design of BRANCH as a Web-based app as it did not take up data space on their phone. At the individual level, Cut-downers experienced strong emotional responses to the app when they achieved a goal of cutting down in comparison with Trackers who felt this way when they successfully entered data for a period. Motivation level also played a large part in their usage of the app as they had a specific goal they wanted to achieve, which encouraged them to use the app more frequently.

##### Barriers to Use for Cut-Downers

At the app level, Cut-downers were not expecting a Web-based app and some experienced Web-based app accessibility issues. However, similar to the Trackers, having high levels of motivation to use the app meant that they were willing to put in effort to learn how to use Web-based apps. Registration issues, such as passwords not working, were cited as barriers. Cut-downers who did not reduce their alcohol-use suggested that lack of reminders contributed to this. Similarly, some struggled to make plans ahead to cut down drinking and highlighted that it was not practical to set goals when they had social occasions planned. This demonstrates the conflict between the functioning of the app in a potentially risky environment of high exposure to alcohol consumption at social events. One participant commented that the social newsfeed was not used frequently enough for it to be engaging. They also commented that the Information and Feedback sections were not regularly updated with new information to make it interesting enough to return. Regarding the social feature, similar to Trackers, Cut-downers were concerned with the privacy element of this component. One participant reported that he disengaged with the app because it helped him achieve his aim of reducing his alcohol consumption, which is a term known as *effective (dis)engagement* [12].

#### The Noncommitter: Lack of Motivation to Use Health Apps

The Noncommitters did not engage with the BRANCH app at all and were all low engagers. They cited a variety of reasons for being attracted to the app in the first place, such as spending, curiosity, health, or understanding their drinking habits, but their initial interest and motivation quickly faded. Although they had good intentions about using the app at first, such as becoming healthier, Noncommitters reported lacking the motivation to even log in to the app, let alone use the app features. They did not regularly use other health apps and would say that this was because they were too *disorganized* or *lazy* to use them:

I didn’t use it for very long because I thought it was too tedious and I wasn't willing to input every kind of drink I had…it was just kind of like a fad thing that I would start doing for a bit and then forget about.

Male, 23 years, low engager

##### How Noncommitters Engaged With BRANCH Features

Noncommitters typically had a quick look about the newsfeed and the drinking diary and perhaps entered a drink or 2 into the diary. Like the other 2 typologies, Noncommitters commented on concerns over the social element. These were primarily about concerns of privacy and about how discussing harmful drinking is not socially acceptable and is still a taboo. Noncommitters did not want other people to find out that they were using the app, with 1 commenting on how using an app such as BRANCH is different from participating in an event such as Dry January, which is a common and an acceptable activity to partake in because it is not assuming that one has a *problem* with alcohol. Noncommitters did not use the other features, such as goals, information, or feedback, lacking the motivation to use them or not even knowing they existed. A few scanned over the features but did not engage with them meaningfully:

I think I added in one drink and I remembered something else I drank, so I went back in and added it in, I haven’t been back since.

Male, 25 years, low engager

##### Impact on Drinking for Noncommitters

Noncommitters did not report any significant impact on their drinking. One participant stated that it may have had a temporary effect, which was quickly lost as he disengaged. In contrast to the other 2 typologies, they did not report the app increasing any awareness in their drinking:

I don’t think it has really affected it much but I just think that’s because I haven’t used it a lot. But I think if I did, it would’ve, because I’d have seen like the amounts that I have been drinking.

Female, 20 years, low engager

##### Facilitators to Use for Noncommitters

As the group that engaged least with the BRANCH app, there were few facilitators to use for the Noncommitters. One participant mentioned that having a Web-based app meant that it did not take up storage on the phone. A few also commented that they appreciated the functionality of the drinking diary and that the graphs were helpful. However, this was not sufficient to encourage them to return to the app.

##### Barriers to Use for Noncommitters

At the app level, participants were expecting a native app and were confused about how to use a Web-based app. There were also barriers in terms of needing internet connection, there being no reminders, and too many steps to input data. This meant they were put-off using the app again. As the individual-level motivation was a major barrier, although initially citing good intentions to want to use the app, these seemed to quickly wane. Noncommitters typically expressed not being *bothered* to go through the various required steps, feeling that it was *too much effort* to input data and having to access the app on the Web. This contrasts to the Trackers and Cut-downers who while experiencing similar issues had the motivation to overcome these barriers and learn how to use the app effectively. This suggests that the level of motivation the Noncommitters had to track and cut down their drinking was significantly lower than the other 2 typologies.

## Discussion

### Principal Findings

Understanding how users interact with and use Web-based and digital health technologies is an important step to improving engagement rates and user experience. The examination of usage patterns can identify typologies, exploring whether there are important differences in how users interact with the app. The major finding of this study was the identification of 3 discreet typologies of engagers to explore the individual experiences of using the BRANCH app: the Tracker, the Cut-downer, and the Noncommitter. To the authors’ knowledge, this is the first study to qualitatively identify a typology of engagers with a smartphone-based alcohol intervention. These data can be used to improve content and functionality of the intervention and to tailor the intervention to the user or certain groups of users, thereby increasing engagement and potentially the effectiveness of the intervention. On the basis of these findings, the key feature to improve BRANCH would be to determine the user typology on app registration, such as via a short questionnaire, and target the app content to their typology.

The Tracker had the largest group of users and was defined by their motivations to use health-tracking apps to monitor and understand quantified self-data. Users did not have intentions necessarily to cut down but purely to track and predominantly used only the drinking diary in the app to enter drinking data. This gave them a sense of control over their lives. They were characterized by frequent usage of a range of health monitoring apps such as fitness, calories, sleep, and spending. Cut-downers were motivated to use the app because they wanted to reduce their alcohol consumption. This group’s users, unlike Trackers, did not use many different health apps but saw the BRANCH app as an opportunity to try to cut down their alcohol use. Similar to Trackers, they not only used the monitoring features of the app but also the goal setting and feedback functions. Noncommitters were characterized as a group of users who were initially enthusiastic about using the app; however, this enthusiasm quickly waned and they only used the app once or twice, gaining no benefit from it. Noncommitters were particularly defined as lacking in motivation to make any behavior changes and were easily put-off in terms of using the app by any usability issues or perceptions that the app required too much time and effort to use.

Although the 3 typologies did occupy discreet categories of engagers, there were similarities between the groups in terms of how they perceived barriers and facilitators to engagement. This is important for future alcohol brief intervention app development as across typologies, barriers and facilitators imply *core* or *fundamental* usability and component issues that need to be considered when designing an app. For example, almost none of the participants were familiar with Web-based apps and cited this as a barrier to use as it required additional effort and time to log in. This is consistent with previous usability testing research [6], which suggests that users want features *at their fingertips*, with minimal required input and effort. Indeed, app-level barriers were the most frequently cited barrier to engagement across the typologies. Issues such as lack of reminders and content updates were highlighted by all typologies. Although improvements were made to the prototype version of the app during its development, clearly, usability is a persistent and enduring issue, which is a priority for optimal eSBI app development.

### Comparisons With Previous Work

No previous qualitative study has explored typologies of user engagement in the alcohol field. Smith et al [15] explored a similar concept of *trajectories of use* with a smoking cessation app and identified 4 different engagement trajectories: (1) productive engagement, (2) counterproductive engagement, (3) productive disengagement, and (4) counterproductive disengagement. Considering this study in light of this conceptual model, Trackers and Cut-downers displayed characteristics of productive engagement and disengagement, being invested in the program and (some of) its components, using adaptive strategies to overcome any barriers of usage and describing disengagement with the app because they achieved their goals of use. Similarly, Noncommitters displayed characteristics of counterproductive engagement and disengagement, having a negative response to the program, not relating to intervention content and disengaging because of usability or motivation factors. Although smoking and alcohol intervention apps often share similar behavior change techniques such as setting goals and monitoring [25], there are differences in the objectives between them. For example, in the smoking domain, cessation is the goal of apps, whereas in the alcohol field, apps typically target hazardous and harmful drinkers, as opposed to dependent drinkers, where reduction not abstinence is the goal. As such, the goals and level of motivation of the app user may differ, which may potentially differentially affect user engagement.

Smith et al [15] focused on identifying trajectories of usage through the program life cycle. This study extends their work by building individual-level factors into the engagement framework, such as personal user characteristics to define engagement not only through usage but also through personality, other app usage, and detailed exploration of motivations and intentions for usage. Subsequently, this study contributes to creating a comprehensive picture of how people engage with eSBI at the individual, environmental, and technological level.

Another similarity across the Tracker and Cut-downer typologies was that most of these users did not report a reduction in alcohol use but instead an increased awareness of level of alcohol consumption and promotion of more *mindful* drinking. Indeed, even for the Cut-downers, when prompted about how they felt when did not achieve their alcohol reduction goal, they constructed this failure with a positive narrative in which they gained awareness, which was achievement enough. This is an important finding because it raises the question of what is the primary goal of eSBI apps and what are the subsequent clinical implications these findings may have. In terms of risk zones for harmful drinking, the app may slow down or stop transitions between zones by identifying at-risk individuals, or it may sit on the pathway between as an identification tool to support patients into treatment. Such steps are critical, as it is important to treat alcohol use in the early stages before it has developed into dependence.

Although a recent Cochrane systematic review examining personalized digital interventions for reducing hazardous and harmful alcohol consumption in community-dwelling populations reported that participants using a digital intervention drank approximately about 3 UK units less than those who received no or minimal interventions; this included all types of digital interventions and was not specific to apps. The evidence for eSBI apps to reduce alcohol consumption is currently inconclusive [2,26-29], and it has been argued that there are differences in the way that eSBI apps may be used by target users compared with eSBIs, which are computer based [6,8]. For example, app-based interventions can be used quickly while on the go, collecting data in the moment; however, computer-based interventions require users to sit down and dedicate time and effort to the program. This may influence the level of the effectiveness of the program or may highlight differences between users. Those who are dedicated to using a computer-based eSBI may have higher levels of motivation to reduce their alcohol use, which would explain why the evidence for eSBI apps is inconclusive. Looking at *effectiveness* from a different perspective, perhaps, the aim of eSBI apps may not be a reduction in units but increased awareness leading to other interventions. For example, Cut-downers may attend their general practitioner (GP) after using the app, and the subsequent GP intervention may reduce their level of drinking.

The lack of use of the social and gamification component was a surprising finding considering these features had been designed in collaboration with the target user group and because previous research has supported the use of such features in the broader health behavior change literature [30,31]. The main reasons cited for nonusage were that (1) they were concerned about privacy issues and (2) cutting down on alcohol use is considered an individual instead of a group activity. Unlike other types of health behavior change such as exercise or weight loss, there exists a persistent social stigma about cutting down on alcohol use; it is, therefore, consistent that participants were wary of using the social component in BRANCH app. The effectiveness of social components in digital alcohol harm reduction interventions is unclear. Although preliminary developmental work for the BRANCH supported the use of social features [8], research on existing smoking and alcohol interventions has suggested that users do not want to share their progress on social networks [25]. A recent systematic review [7] of RCTs that examined the effectiveness of EPSs in Web-based substance misuse interventions reported ambiguous outcomes for the use of social features. It may be that social features need to be adapted further for substance-use interventions focusing on establishing trust about privacy and targeting issues of stigma about cutting down on alcohol use.

In terms of implications for future design of eSBI apps, based on the current research, it is recommended that push notifications or SMS reminders are used. Overall, users were unfamiliar with Web-based apps, and native apps may be a more appropriate user platform. Frequently updated information and improved usability should also be considered in future eSBI app design.

Tailoring is also an important feature for improvement of eSBI apps. A previous study [7] highlighted tailoring to self-efficacy as potentially effective, as well as some support for tailoring to motivations for quitting, abstinence status, and a personalized source. This qualitative study suggests that tailoring to engagement typology might also be associated with improved outcomes. Future iterations may benefit from creating an app where certain features could be turned on and off, tailoring features to different typologies. For example, Trackers might benefit most from an eSBI app that prioritized optimization of the monitoring features such as having the drinking diary as the home page and more sophisticated data visualizations. Cut-downers may need an app that is targeted to supporting users reach their goals, for example, more emphasis on positive reinforcement of goal completion and tangible milestones for cutting down integrated into the app. Nonengagers might need an app with features to enhance motivation to engage with behavior change as a precursor to further app usage.

This study questions how we measure the *effectiveness* of eSBI apps. The objective of eSBI apps may not be to reduce alcohol consumption but to serve as a tool to support people to seek other types of interventions and treatment. Perhaps eSBI is not a stand-alone treatment for reducing alcohol use (as opposed to computer-based eSBI) but is in fact a low-intensity tool to facilitate harmful drinkers to become aware of their level of drinking and be a step in the treatment pathway toward behavior change. From a clinical perspective, such apps could be prescribed by clinicians to patients to make them aware of the level of their drinking, and then more intensive treatments such as computer-based interventions could be recommended. However, as the first study to report such findings, this needs further investigation before absolute recommendations can be made.

Another question that this study raises is whether eSBI apps are a suitable intervention for everybody or whether they are an appropriate intervention for certain typologies. Trackers and Cut-downers both reported benefits from the program although from an awareness rather than a reduction perspective. However, Noncommitters did not report any tangible benefit. On the one hand, it may be that certain individuals enjoy using health monitoring apps and are more likely to see a positive effect. Others, such as the Noncommitters, do not enjoy entering data into apps and find it difficult to engage with such programs and, therefore, may benefit from more traditional face-to-face interventions or apps that aim to enhance motivation to engage in further interventions or behavior change. From a clinical perspective, it may be that there is not a *one-size-fits-all* solution to alcohol brief interventions (BIs), and patients should be offered a range of tools, including digital and face-to-face interventions, to select what works best for them and their lifestyle.

On the other hand, it is also plausible that there is scope within eSBI to target motivation levels within individuals who do not initially engage, such as the Noncommitters. The findings of the study suggest that because different typologies have different levels of motivation to cut down, eSBI may be compatible with the application of the Transtheoretical Model of Change [32] to increase motivation level to cut down. For example, the app could be tailored to stage of change, such as through a questionnaire at registration stage, followed by specific intervention components tailored to the user’s motivation level. However, further research is needed to explore whether or not this would be an effective feature. It is suggested that future research examines further engagement from the perspective of the Transtheoretical Model of Change. With future enhancements in technology and the potential linkage of health to digital health watches such as Fitbits, it is plausible that such devices could measure blood alcohol concentration (BAC) transdermally, eradicating the need for self-report input and many of challenges faced by current eSBI. In the future, apps in conjunction with automated BAC calculations may be able to automatically measure usage and provide tailored support and advice without the need for human input.

### Limitations

The majority of participants were female (16/20, 80%). Although potentially introducing bias, this is consistent with previous research published on the BRANCH app where 90% of the qualitative sample was female [6,18]. This is also consistent with the characteristics of participants of the main RCT, with a 70% female sample. Research suggests that women are more likely to use Web-based resources to access health information [33,34]. As such, this sample may reflect the type of individual most likely to engage with eSBI interventions. Future studies may wish to explore in more detail why females are more likely to engage and examine how eHealth interventions can be tailored to gender.

Recruitment may have discouraged potential participants who had negative experiences or limited engagement with the app. Some potential participants contacted declined to participate as they felt they did not have enough feedback to offer because of low engagement. However, efforts were made to outline that participants who did not use the app frequently were still invited to participate; subsequently, an equal split of high and low engagers were recruited. Qualitative interviews can be subject to response bias where participants provide views that they believe the researcher wants to hear. A couple of *nonengagers* (logged in once), identified from app-usage data extracted from the server, did report using the app more than once. This may imply a response bias, or poor memory recall, and may have resulted in some participants over-reporting app usage, potentially biasing the analysis. However, a strength of the study overall was that it recruited both high and low engagers, so a range of views was provided. Characteristics of trial participants who declined to participate in the qualitative interviews were not collected. Therefore, there may exist a bias in the characteristics of participants recruited, such as the high-engaging participants being more motivated to provide positive experiences of using the app than low engagers who declined to participate. All participants were selected as harmful drinkers (Alcohol Use Disorder Identification Test (AUDIT) score 16+). As alcohol is still considered a stigmatizing subject, participants may have not shared all their experiences of drinking at a harmful level. Efforts were made by the researcher to provide a nonjudgmental space in which to discuss their experiences of drinking. The data were analyzed by only a single researcher, which may have biased the results. As this is the first study in the alcohol field to qualitatively define typologies of user engagement, the generalizability of the findings requires further research. However, as the findings are consistent with quantitative work [35,36] and qualitative work from the smoking cessation field [15], this suggests the findings are generalizable to the broader population.

### Conclusions

This study has identified 3 typologies of eSBI app users. Trackers use only monitoring features and are not interested in cutting down, only measurement; Cut-downers use the app to reduce their alcohol use and will use more features such as goal setting functions; and Noncommitters have good intentions but quickly disengage from using the app. Although in need of replication, it provides a first step in understanding how eSBI apps can be tailored to different user types to improve engagement and ultimately effectiveness. It also questions what the purpose or utility of eSBI apps is. As opposed to reducing consumption, eSBI apps may serve as a tool to provide a stepping stone in the pathway of treatment to prevent individuals developing more serious alcohol-related conditions. With the consistent findings from eSBI app trials that apps may not be as effective as computer-based methods as previously thought, perhaps it is time to rethink how we conceptualize the purpose and function of eSBI apps in the future.

## Appendix

Multimedia Appendix 1 Topic guide.

Multimedia Appendix 2 Matrix of engagement typologies.

## Abbreviations

BAC: blood alcohol concentration
CMUE: conceptual model of user engagement
eHealth: electronic health
EPS: engagement-promoting strategy
eSBI: electronic screening and brief intervention
FRAMES: Feedback, Responsibility, Advice, Menu of options, Empathy, Self-efficacy
GP: general practitioner
NIHR: National Institute for Health Research
NHS: National Health Service
PSD: Persuasive Systems Design
RCT: randomized controlled trial
SMS: short message service
