# Supplementary material for: Developing Typologies of User Engagement With the BRANCH Alcohol-Harm Reduction Smartphone App: Qualitative Study
Source: JMIR Mhealth Uhealth. 2018 Dec 13;6(12):e11692. doi: 10.2196/11692 (PMC6315270; doi:10.2196/11692)
Supplement: Multimedia Appendix 1 [file mhealth_v6i12e11692_app1.pdf]

Topic Guide V1 19/04/16

Introduction

Thank you for agreeing to speak with me today. I am interested in your experience of using the app, good and bad.

- Tell me a bit about yourself?

Signing up to the trial

Think back to when you first saw the advert for the app… tell me about what your thoughts were?

- Why did you decide to download the app?
- Why did you want to participate in the study?
- How did you think the app might be useful to you?

Registration process

Now, think specifically about the process of registering for the app… after you received the SMS link. Can you describe this process step by step?

- What did you think about the registration process?
- Any problems logging in to the app?
- Did you find anything confusing?

Low engager

- Tell me everything you remember about using the app? What did you do?
  - Goals
  - Team
  - Information
  - Newsfeed
  - Feedback
  - Drinking diary

- Tell me about how you felt when you used the app? Is there anything you particularly enjoyed? Didn't enjoy?

- How would you describe the app to a friend? What would you tell someone about using it who hadn't seen it before?

- Why did you stop using the app?

High engager

- Think back to when you first started using the app, what did you use it for?

- Has use of the app changed over the time? Please describe
  - How did it change?
  - Why did it change?
  - Did you use it more or less?
  - What was the last thing you used the app for?

- I would like you to think about a time where you were enjoying using the app. Please tell me about this?

- Now think of a time where you didn't enjoy using the app.
  - Why was this?
  - When?
  - Under which circumstances?

- How would you describe the app to a friend? What would you tell someone about using it who hadn't seen it before?

- Why did you stop using the app?

Impact on life and drinking

Tell me how, if in any way, your life is affected as a result of using the app?

- How would you describe the impact on your drinking?

- How do you think the app might help someone cut down their alcohol use?

- Has it helped you? Has it made things worse? Have you felt discomfort when using the app? Has using the app made you seek medical care?

Improvement of the app

- How do you think the app could be improved? Design, features, usability?
- Were there any features you particularly liked/disliked?

Use of other apps

Tell me about your favourite app on your phone?

- How do you use it?
- Why do you like it?
- How does this app compare to the BRANCH app?

What are your opinions on using apps to help you keep fit and healthy?

Tell me about how you keep fit and healthy

End

Do you feel there was anything or any question that was not covered that you would like to talk about?

Do you have any questions for me?

Thank you for taking part.
