# Supplementary material for: Developing Typologies of User Engagement With the BRANCH Alcohol-Harm Reduction Smartphone App: Qualitative Study
Source: JMIR Mhealth Uhealth. 2018 Dec 13;6(12):e11692. doi: 10.2196/11692 (PMC6315270; doi:10.2196/11692)
Supplement: Multimedia Appendix 2 [file mhealth_v6i12e11692_app2.pdf]

| | Motivation for participation | Primary feature usage | Facilitators to use | Barriers and disengagement | Impact on drinking | Other app usage |
|---|---|---|---|---|---|---|
| **Tracker** | (1) Track drinking patterns<br>(2) Track health or weight<br><br>(3) Track spending<br><br>(4) Awareness of drinking habits<br><br>(5) Curiosity | (1) Used drinking diary only<br><br>(2) Consistent usage<br><br>(3) Additional features largely ignored. | *App level:*<br><br>(1) Main facilitator to use were features that enabled efficient entering of data<br><br>(2) Accuracy of data input<br><br>(3) Visualization of trends<br><br>*Individual level:*<br><br>(4) Positive emotional experiences engaged | *App level:*<br><br>(1) Web app or usability or not intuitive or no reminders<br><br>(2) Too many steps to input data<br><br>(3) No updates<br><br>(4) Goals too 'abstract'<br><br>(5) Info not targeted or relevant or needed sources | (1) Increased awareness of drinking patterns, made more 'mindful'<br><br>(2) Few referenced a small change in behavior of 'not having that second glass of wine' | Lots of apps to track all aspects of health and lifestyle |

| | | | | | | |
|---|---|---|---|---|---|---|
| | | | by<br><br>tracking features increased motivation and engagement e.g. *Empowerment, control, security* | ***Environmental:***<br><br>*(6)* Too tired or busy or went on holiday etc. | | |
| **Cut-Downer** | 1) Cut-down drinking<br><br>2) Track<br><br>3) Spending<br><br>3) Health<br><br>4) Awareness | 1) All high engagers<br><br>2) Typically used drinking diary, goal and feedback component s<br><br>3) No use of teams or | ***App level:***<br><br>1) Ease of use<br>2) Web app did not take up storage on phone<br>3) Info on risks and costs 'eye opening'<br><br>***Individual level:***<br><br>4) Positive emotional experiences engaged by goal and cutting | ***App level:***<br><br>1) web app or usability or no reminders<br><br>2) Drink accuracy- brands or cocktails<br><br>3) No updates of material<br><br>4) Goals not positively reinforcing | 1) A few reports of cutting down<br><br>2) Reports of increased awareness of drinking patterns, made more ‘mindful’ | No |

| | | | | | | |
|---|---|---|---|---|---|---|
| | | social component | down features: elicited responses of empowerment | enough and not tangible enough with milestones<br><br>***Individual level:***<br><br>5) App started to work | | |
| **Non-Committer** | ***Mixed reasons no consistency:***<br><br>1) Spending<br><br>2) NY resolution<br><br>3) Health<br><br>4) Cut down | 1) Non engagers<br><br>2) Put in a drink or two<br><br>3) Didn't engage with features, frequently didn't even know they | ***App level:***<br><br>1) Storage not taken up on phone<br><br>2) Easy put in drinks<br><br>3) Liked visualizations | ***App level:***<br><br>Usability issues a major reason for disengagement or not native or no reminders<br><br>***Individual level:***<br><br>Motivational. Too much effort, not | No significant Impact | A few health apps for a short amount of time |

| | | | | | | |
|---|---|---|---|---|---|---|
| | 5) Curiosity | were there | | bothered, too many steps | | |
